# Diagnostic accuracy and reliability of susceptibility map-weighted imaging in patients with clinically uncertain parkinsonian syndromes

**DOI:** 10.1186/s41747-026-00832-w

**Published:** 2026-11-14

**Authors:** Elon D. Wallert, Martijn Beudel, Vera C. Keil, Esther Sánchez Aliaga, Frederik Barkhof, Tom van Mierlo, Jeroen Blankevoort, Henk W. Berendse, Rob M. A. de Bie, Jan Booij, Elsmarieke van de Giessen

**Affiliations:** 1 Department of Radiology and Nuclear Medicine, Amsterdam UMC, location Universiteit Amsterdam, Amsterdam, The Netherlands; 2 Department of Neurology, Amsterdam UMC, location Universiteit Amsterdam, Amsterdam, The Netherlands; 3 Department of Radiology and Nuclear Medicine, Amsterdam UMC, location Vrije Universiteit Amsterdam, Amsterdam, The Netherlands; 4 Queen Square Institute of Neurology and UCL Hawkes Institute, University College London, London, UK; 5 Department of Neurology, Spaarne Gasthuis Hospital, Haarlem, The Netherlands; 6 Department Neurology, Flevoziekenhuis Hospital, Almere, The Netherlands; 7 Department of Neurology Amsterdam UMC, location Vrije Universiteit Amsterdam, Amsterdam, The Netherlands

**Keywords:** Image interpretation (computer-assisted), Magnetic resonance imaging, Parkinsonian disorders, Sensitivity and specificity, Substantia nigra

## Abstract

**Objectives:**

Parkinson’s disease (PD) is characterized by degeneration of dopaminergic neurons in the substantia nigra, accompanied by increased iron accumulation, particularly in the nigrosome-1 subregion. Differentiating neurodegenerative disorders like PD from non-neurodegenerative causes of parkinsonism can be challenging. In clinically uncertain parkinsonian syndrome (CUPS), dopamine transporter (DAT)-single-photon emission computed tomography (SPECT) can aid diagnosis. However, susceptibility map-weighted imaging (SMWI) provides a promising magnetic resonance imaging (MRI) alternative. We evaluated the diagnostic accuracy and reliability of visual SMWI assessment by radiologists in CUPS and the added value of artificial intelligence (AI)-driven diagnostic software.

**Materials and methods:**

Patients with CUPS who underwent DAT-SPECT between January 2019 and July 2023 were included. Four radiologists, blinded to clinical symptoms and diagnosis, visually rated nigrosome-1 on axial SMWI reconstructions as normal, unclear, or abnormal. After the initial assessment, the radiologists re-evaluated the scans with AI-driven software assistance, designed to classify and quantify nigrosome-1 integrity. The reference standard was the final clinical diagnosis incorporating DAT-SPECT results.

**Results:**

One hundred eighty patients were included (mean age 67.9, range 31–86, 120 males), 84 with neurodegenerative and 96 with non-neurodegenerative parkinsonism. Mean diagnostic accuracy was 80% (range 73–83%), with substantial inter-rater reliability (Fleiss κ 0.65). With software assistance, diagnostic accuracy improved to 87% (range 85–91%), and inter-rater agreement increased to almost perfect (Fleiss κ 0.86).

**Conclusion:**

SMWI, particularly with AI-driven software assistance, is a promising and accessible MRI-based technique that may support the diagnostic workup in clinically uncertain parkinsonism, aiding the distinction between neurodegenerative and non-neurodegenerative causes.

**Key Points:**

***Question*** Can susceptibility map-weighted imaging (SMWI) reliably differentiate neurodegenerative from non-neurodegenerative parkinsonism in clinically uncertain cases, and does artificial intelligence (AI)-driven software assistance improve radiologist performance?

***Findings*** Visual assessment had an accuracy of 80%, with substantial inter-rater reliability. AI-driven software assistance increased accuracy to 87% and improved inter-rater reliability.

***Relevance statement*** SMWI can differentiate neurodegenerative from non-neurodegenerative parkinsonism in clinically uncertain cases and may support the diagnostic process by offering an accessible MRI-based technique, facilitating an efficient diagnostic workup, and enabling early diagnosis in clinically uncertain parkinsonism.

**Graphical Abstract:**

**Figure:**
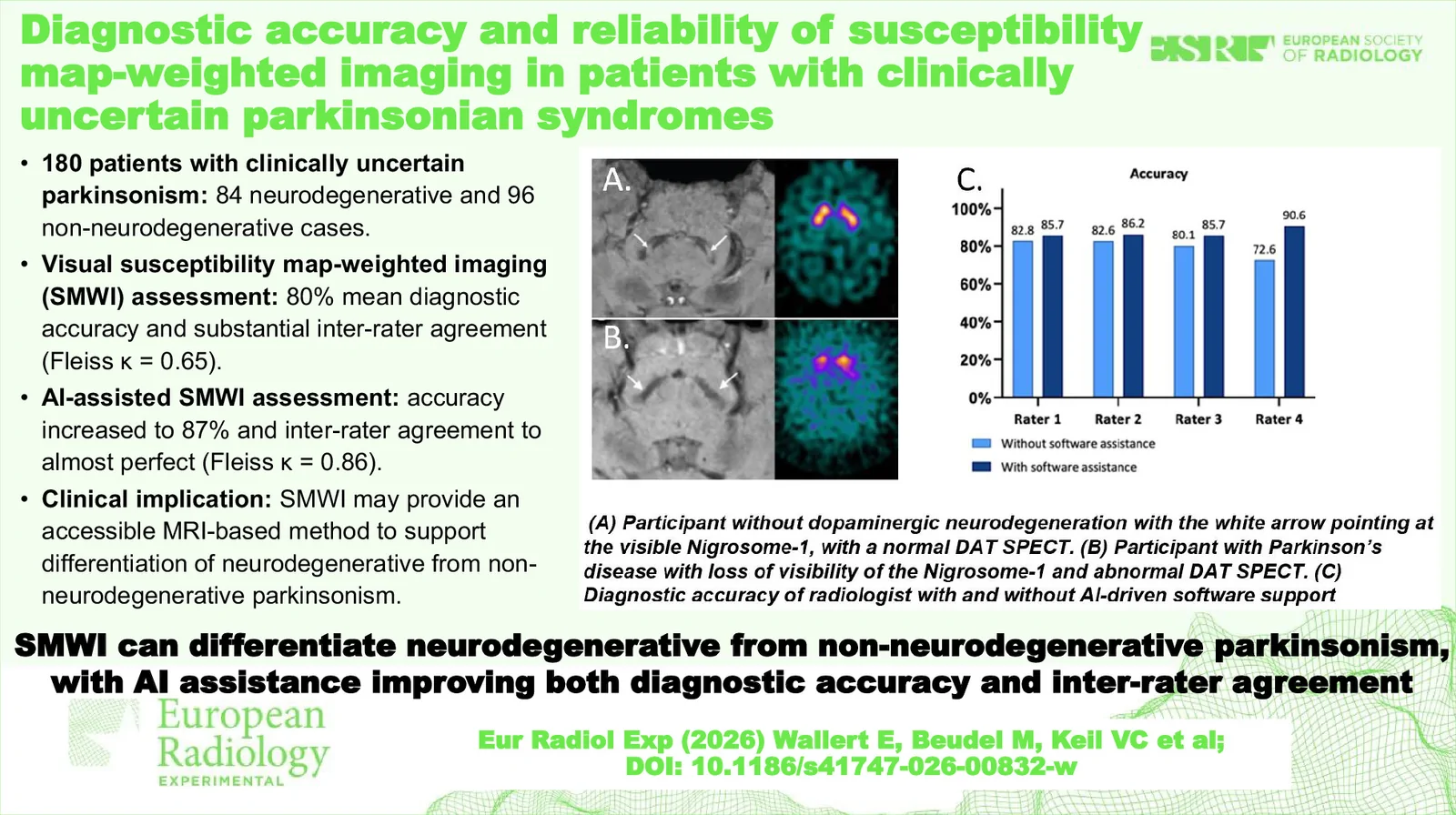

## Introduction

Diagnosing Parkinson’s disease (PD) can be challenging in clinical practice. In the diagnostic process, it is essential to correctly differentiate between neurodegenerative diseases like PD and non-neurodegenerative causes of the parkinsonian symptoms, *e.g*., drug-induced parkinsonism. PD is characterized by dopaminergic neural loss in the substantia nigra (SN), leading to motor and non-motor symptoms. Generally, the diagnosis is based on the anamnesis and neurological examination; however, in some patients, the diagnosis remains inconclusive. These cases can be classified as clinically uncertain parkinsonian syndromes (CUPS). To aid in the diagnosis of CUPS, dopamine transporter (DAT) imaging with single-photon emission computed tomography (SPECT) or positron emission tomography (PET) can help to accurately distinguish neurodegenerative from non-neurodegenerative causes [1].

Most patients with CUPS undergo magnetic resonance imaging (MRI) before nuclear imaging. In current clinical practice, however, MRI is generally limited to conventional sequences and primarily used to rule out alternative causes of parkinsonism, such as vascular lesions, rather than to diagnose neurodegenerative parkinsonism. Since MRI scans are widely available, an MRI-based method to aid in the diagnosis of PD would significantly improve clinical practice. From this perspective, the novel Susceptibility Map-Weighted Imaging (SMWI) sequence has been developed. SMWI enhances susceptibility contrast by combining magnitude images with a quantitative susceptibility mapping (QSM)-based weighting factor [2, 3]. This enables visualization of the cell-dense nigrosome-1 subregion in the SN. In patients with neurodegeneration, nigrosome-1 is no longer visible due to neuronal loss and locally increased iron deposition [4–6]. Unlike conventional susceptibility-weighted imaging (SWI), which combines magnitude and phase information, the voxel intensities in QSM directly reflect tissue magnetic susceptibility and correlate with iron content [7–9]. In a comparative study, SMWI demonstrated a higher contrast-to-noise ratio in the nigrosome-1 region than QSM alone in healthy controls [2]. Another study comparing SMWI and SWI reported a higher diagnostic accuracy for visual assessment using SMWI (area under the receiver operating characteristic curve 0.79 *versus* 0.72) [10].

Previous studies reported on the diagnostic accuracy of SMWI to distinguish patients with PD from healthy controls. The accuracy of visual assessment ranged from 71.7% to 97.7% [10–13]. Automated artificial intelligence (AI)-driven software analysis yielded an area under the receiver operating characteristic curve of 0.92 to 0.97 [13–15]. However, these studies were performed in patients with an already established diagnosis rather than in patients with CUPS, for whom improving diagnostic certainty would be most relevant. Hence, the diagnostic accuracy of SMWI remains unclear in this clinically relevant population, along with the inter-rater-agreement and the possible effect of AI software assistance on diagnostic performance.

This study aimed to evaluate the diagnostic value of visual SMWI assessment in patients with CUPS, both with and without AI software support. We hypothesized that SMWI has high diagnostic accuracy for detecting neurodegenerative parkinsonism in this population and that software assistance further improves performance. Additionally, we expected that inter-rater agreement would increase with software support, enhancing diagnostic consistency. These findings would support the role of SMWI in clinical practice for CUPS patients, possibly reducing the need for DAT imaging in the future.

## Materials and methods

**Fig. 1:**
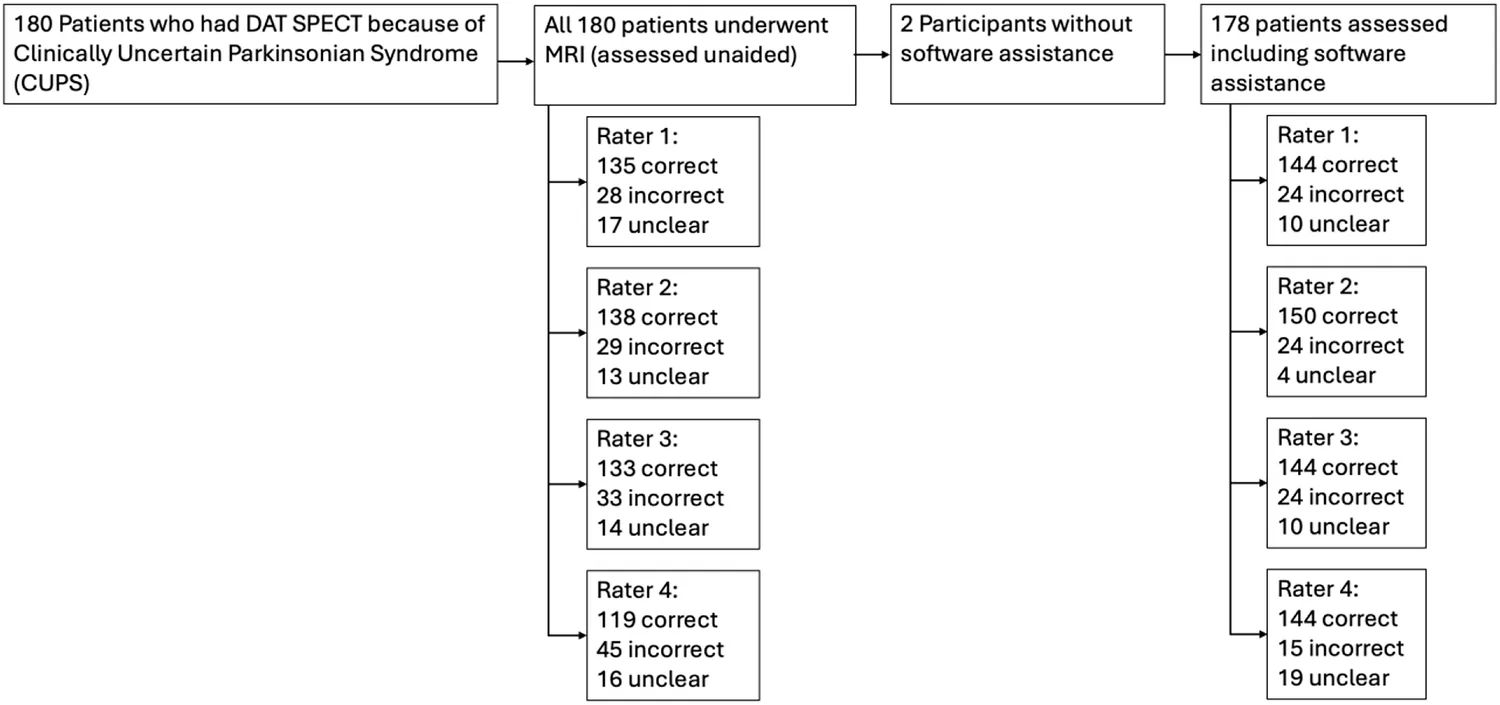
Flow diagram of included patients and number of correct, incorrect, and unclear results per rater, without and with AI support

The study was approved by the Amsterdam UMC institutional review board (9240.018.22). All participants provided written informed consent. We conducted a diagnostic evaluation study in patients with CUPS. We retrospectively identified and approached consecutive eligible patients. After inclusion, participants underwent an MRI scan (Fig. 1). Details of patient inclusion have been described previously [16].

### Population

We included patients above the age of 18 years, who presented with parkinsonism at the neurology outpatient clinics of the Amsterdam University Medical Center, and two other hospitals (Spaarne Gasthuis and Flevoziekenhuis hospitals), and had a DAT (^123^I-fluoropropyl-carbomethoxy-iodotropane (FP-CIT))-SPECT because of CUPS after January 1, 2019.

Patients were considered to have CUPS when the treating neurologist, taking into account medical history (including use of medications) and neurological examination, could not confidently distinguish neurodegenerative from non-neurodegenerative parkinsonism and therefore requested DAT-SPECT imaging. The reference diagnosis was the final clinical diagnosis documented during follow-up, integrating the DAT-SPECT result and the clinical course; the cohort therefore represents patients who were clinically uncertain at referral, with diagnostic certainty increasing over follow-up to varying degrees. Patients unable to provide informed consent or with contraindications for MRI were excluded from the study.

A power analysis showed that a sample size of 180 participants would allow an estimate of sensitivity and specificity with a precision of 6.2%, with a 95% confidence level, assuming a prevalence of neurodegeneration as the cause of the parkinsonism in half of the participants, and a sensitivity and specificity of 90%.

### Index test and assessment

**Fig. 2:**
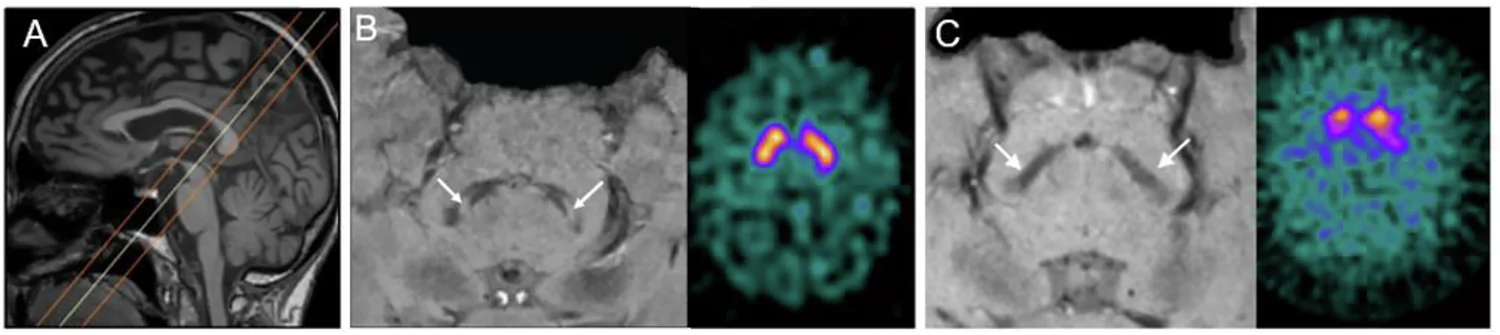
Representative images of (**A**) planning of SMWI slices, coronal-transversal oblique plane perpendicular to the mesencephalon, covering the posterior commissure to the top of the pons, (**B**) participant without neurodegeneration with white arrows pointing at the visible nigrosome-1, with a normal DAT-SPECT, (**C**) participant with PD with loss of visibility of nigrosome-1 and an abnormal DAT-SPECT. DAT-SPECT, Dopamine transporter single-photon emission computed tomography; PD, Parkinson’s disease; SMWI, Susceptibility map-weighted imaging

All participants had an MRI scan of the brain at the Amsterdam UMC on the same 3-T Ingenia MRI system (Philips Healthcare) with a 32-channel head coil, including a magnetization prepared rapid acquisition with gradient-echo–MPRAGE three-dimensional T1-weighted sequence for planning the SMWI. The multiecho three-dimensional fast field echo SMWI sequence (field of view 192 × 192 × 32 mm^3^, acquisition matrix 384 × 384, voxel 0.5 × 0.5 × 1 mm^3^, 32 slices, repetition time 49.0 ms, first echo time 14 ms, echo spacing 13.0 ms, 3 echoes, flip angle 20°, receiver bandwidth 100 Hz/pixel, acquisition time 4:55 min:s) was positioned to include the SN in a coronal–transverse oblique plane perpendicular to the mesencephalon, covering the posterior commissure to the top of the pons (Fig. 2).

The index test was the visual assessment of the SMWI sequence as normal or abnormal based on the visibility of the nigrosome-1 subregion. Assessment was done by four radiologists (V.K., E.S., F.B., E.G.), with experience in neuroradiology ranging from 8 to 30 years. The four radiologists were blinded to all clinical information, the final clinical diagnosis, the DAT-SPECT result, and all other imaging findings; they assessed only the SMWI reconstructions. Raters were provided with 20 SMWI training scans. Subsequently, they assessed the scans of the participants on a scoring form with the consecutive SMWI images, rating both left and right nigrosome-1 on a 5-point Likert scale as normal, likely normal, unclear, likely abnormal, or abnormal (supplemental figures). The quality of the scan was rated as very poor, poor, sufficient, or good. After the initial assessment, the radiologists rated 50 scans a second time to assess intra-rater agreement. The order of this case set was randomly selected but identical for all readers. After the first assessment of the scans, they were provided with the results of AI software that classified the nigrosome-1 as normal or abnormal, and provided volumetric measurement (Heuron IPD version 1.0.1.9 and Heuron NI version 1.0.0.18, Heuron Co., Ltd). The radiologists first assessed all cases unaided, and the complete set was then reassessed with software assistance, performed as two separate passes over the entire series rather than case by case, without a defined washout interval between sessions. The IPD software was used for the consecutive reconstruction of QSM and SMWI [2, 3, 14, 15]. The software identifies the red nucleus on SMWI and analyzes the five slices below the inferior pole of the red nucleus (based on the field of view), which include the SN with the presence of nigrosome-1. A trained convolutional neural network (YOLOX) was utilized to assess the nigrosome-1 area and classified the nigrosome-1 as normal or abnormal. In addition, the NI software automatically outlines the nigrosome-1 and provides a volumetric measure. A single, fixed software version was used for all scans throughout the study, and this version was not trained on scans included in this study. Readers integrated these outputs with their own visual assessment, with the final rating left to the reader’s discretion. To assess whether the interval between DAT-SPECT and MRI affected diagnostic performance, we performed an analysis stratifying patients into those scanned ≤ 1 year and > 1 year after DAT-SPECT.

### Reference test

The reference test was the final diagnosis documented in the electronic health record by the treating neurologists, incorporating the DAT-SPECT scan results. In case the diagnosis was not clearly stated, we either inquired with the treating neurologist or the case was reviewed by two movement disorders experts (M.B., R.B.), and a consensus was reached to classify a case as neurodegenerative or not neurodegenerative. DAT-SPECT was performed with ^123^I-FP-CIT. Acquisition and visual interpretation were performed according to the European Association of Nuclear Medicine practice guideline for dopaminergic imaging in parkinsonian syndromes [17]. Scans were interpreted as normal or abnormal by visual inspection, also taking into account center-specific binding ratios. Because patients were referred from several centers over a multi-year period, scans were acquired on different gamma-camera systems, so quantitative specific binding ratios were not comparable across the cohort. Inconclusive scans were reviewed by two blinded, experienced nuclear medicine physicians (J.B., E.G.), who reached consensus to classify the DAT-SPECT as normal or abnormal.

### Clinical data

Clinical data, including medical history and medication use, were extracted from the electronic health record. Symptom severity was assessed using clinical symptom rating scales and questionnaires. The Movement Disorder Society Unified Parkinson’s Disease Rating Scale (MDS-UPDRS) was used to assess the severity of parkinsonian symptoms [18]. The MDS-UPDRS consists of four parts: (I) Non-Motor Aspects of Experiences of Daily Living (range 0–52), (II) Motor Aspects of Experiences of Daily Living (range 0–52), (III) Motor Examination (range 0–132), and (IV) Motor Complications (range 0–24), where higher scores imply more severe symptoms. The Hoehn and Yahr scale assesses disability associated with PD, and higher stages (range 0–5) indicate greater disability [19]. Depressive symptoms were assessed using the Beck Depression Inventory II (BDI), with higher scores indicating more severe depressive symptoms (range 0–63) [20]. The Montreal Cognitive Assessment (MoCA) scale was used to screen for cognitive problems, where the maximum score is 30, and lower scores indicate greater cognitive impairment [21].

### Statistical analysis

Statistical analyses were performed with SPSS (version 30, IBM). Group differences were assessed with a *t*-test for normally distributed data or the Mann–Whitney *U* test otherwise. Diagnostic accuracy, sensitivity, specificity, positive predictive value, and negative predictive value were calculated against the reference standard, with scans rated as unclear excluded but reported separately, and are presented with 95% confidence intervals calculated using the Wilson score method. Differences in diagnostic accuracy with and without software support were tested with the McNemar test. Analyses were repeated after excluding scans of insufficient quality. Intra-rater agreement was assessed with Cohen κ and inter-rater agreement with Fleiss κ. A *p*-value < 0.05 was considered significant.

## Results

### Population

A total of 180 participants were included, of whom 84 and 96 received a final diagnosis of neurodegenerative or non-neurodegenerative parkinsonism, respectively. The mean age was 67.9 years (range 31.9–86.4 years). The sample consisted of 120 men and 60 women. The average interval between the DAT-SPECT and MRI scans was 1.8 years (range 0.1–4.5 years). There were no significant differences in demographic characteristics between the two groups (Fig. 1 and Table 1).

**Table 1:** Patient characteristics and results of the clinical questionnaires and symptom assessment scales

	Neurodegenerative (*n* = 84)	Non-neurodegenerative (*n* = 96)	*p*-value
Age (years) ± SD	67.0 ± 10.2	68.7 ± 10.2	0.27
Sex (female/male)	24 (29%)/60 (71%)	36 (37%)/60 (63%)	
Time between index and reference test (years)	1.90 ± 1.22	1.78 ± 1.16	0.50
MDS-UPDRS I (mean ± SD, *n* = 75/86)	11.4 ± 6.5	13.7 ± 6.8	0.03
MDS-UPDRS II (*n* = 75/86)	9.6 ± 6.7	11.2 ± 7.0	0.13
MDS-UPDRS III (*n* = 74/85)	24.9 ± 12.8	27.7 ± 11.8	0.16
HY 0	1	4	
HY 1	6	1	
HY 2	53	49	
HY 3	12	33	
HY 4	2	3	
HY 5	1	0	
BDI (mean ± SD, *n* = 72/86)	9.8 ± 7.7	11.57 ± 8.5	0.18
MoCA (mean ± SD, *n* = 74/87)	24.0 ± 4.7	23.6 ± 3.9	0.51

*BDI* Beck’s depression inventory, *HY* Hoehn and Yahr, *MDS-UPDRS* Movement Disorder Society Unified Parkinson’s Disease Rating Scale, *MoCA* Montreal Cognitive Assessment

Among 84 patients with neurodegenerative parkinsonism, 76 were ultimately diagnosed with PD. Final diagnoses by group are shown in Table 2. The group labeled “unclassified non-neurodegenerative parkinsonism” comprised patients with a normal DAT-SPECT who did not meet criteria for a specific non-neurodegenerative diagnosis, such as drug-induced parkinsonism or essential tremor.

**Table 2:** Per-group diagnoses of the participants with a clinically uncertain parkinsonian syndrome

	Neurodegenerative		Non-neurodegenerative	
Number of patients	84		96	
Diagnosis (*n*)	Parkinson’s disease	76	Tremor	27
	Multiple system atrophy—parkinsonian type	1	Functional neurological disorder	11
	Multiple system atrophy—cerebellar type	1	Drug-induced parkinsonism	13
	Corticobasal syndrome	2	Vascular parkinsonism	5
	Dementia with Lewy bodies	2	Psychiatric disorder	3
	Unclassified	2	Structural brain abnormality	6
			Unclassified non-neurodegenerative parkinsonism	31

Regarding the clinical questionnaires and symptom severity assessment, only the total MDS-UPDRS I score differed significantly between groups, with a slightly higher mean score in the non-neurodegenerative group (13.7 *versus* 11.4, *p* = 0.03). No significant differences were observed for the MDS-UPDRS parts II and III, MoCA, or BDI-II scores (Table 1).

### Visual assessment

**Fig. 3:**
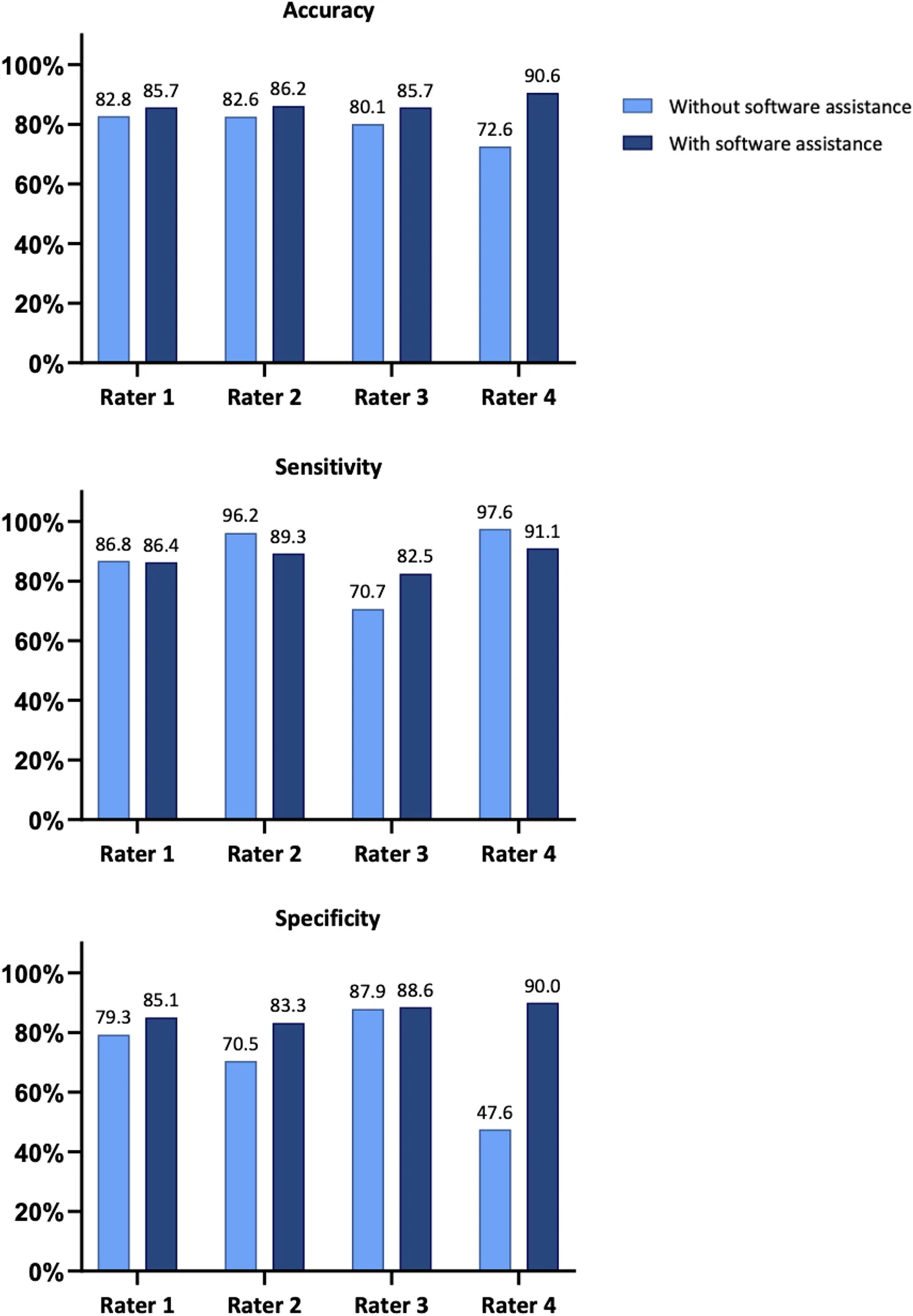
Bar chart of the diagnostic accuracy, sensitivity, and specificity per rater, with and without the availability of the AI diagnostic software assistance

**Fig. 4:**
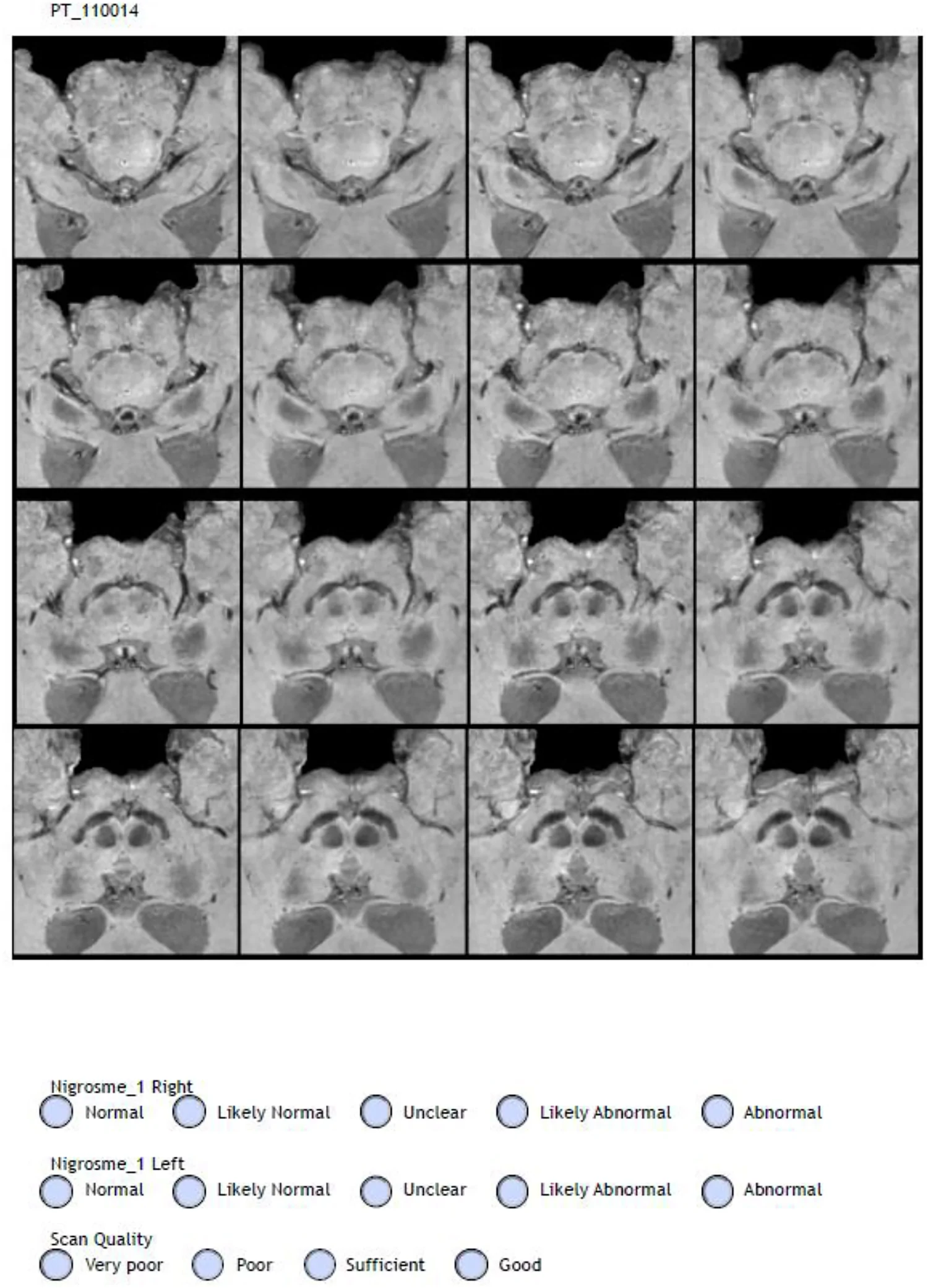
Example of the rating form with the consecutive SMWI images and a 5-point Likert scale, with the options of normal, likely normal, unclear, likely abnormal, or abnormal. The quality of the scan was rated as very poor, poor, sufficient, or good. SMWI, Susceptibility map-weighted imaging

Image quality, as rated by the four radiologists, was judged on average as very poor in 6.0% of the scans (range 2.2–8.9%), poor in 14.7% (range 8.9–20.6%), sufficient in 37.6% (range 31.7–45.0%), and good in 41% (range 29.4–56.1%). On average, 8.3% of the scans were rated as ‘unclear’ (range 7.2–9.4%). The mean diagnostic accuracy across raters was 79.5% (range 72.6–82.8%), with a mean sensitivity of 87.8% (range 70.7–97.6%) and specificity of 71.3% (range 47.6–87.9%) (Table 3 and Fig. 3). When scans with a quality rated as very poor or poor were excluded from the analysis, accuracy improved slightly to 81.3% (range 75.6–83.7%). In the interval-stratified sensitivity analysis, mean unaided accuracy was 83.1% (range 75.6–87.5%) in patients scanned ≤ 1 year after DAT-SPECT and 78.7% (range 74.1–83.0%) in those scanned > 1 year later; the difference was not significant for any reader (*p* ≥ 0.15). To assess intra-rater agreement, three of the four raters re-evaluated a subset of 50 scans. Cohen κ values ranged from 0.63 to 0.95, indicating substantial to almost perfect agreement. The percentage of scans for which the raters changed their assessments ranged from 2% to 19.0%. The inter-rater agreement was substantial with a Fleiss κ of 0.65 (95% CI 0.58–0.72). Pairwise agreement ranged from moderate to substantial (Cohen κ range 0.42–0.74) across rater combinations (Table 4 and Fig 4).

**Table 3:** Diagnostic accuracy, without/with the availability of AI-driven software assistance per rater

	Rater 1		Rater 2		Rater 3		Rater 4	
	Unaided	Software-assisted	Unaided	Software-assisted	Unaided	Software-assisted	Unaided	Software-assisted
Counts (*n*)								
True positive	66	70	76	75	53	66	80	72
False negative	10	11	3	9	22	14	2	7
True negative	69	74	62	75	80	78	39	72
False positive	18	13	26	15	11	10	43	8
Non-diagnostic/missing	17	12	13	6	14	12	16	21
Diagnostic performance, % (95% CI)								
Accuracy	82.8 (76.3–87.8)	85.7 (79.6–90.2)	82.6 (76.2–87.6)	86.2 (80.3–90.6)	80.1 (73.4–85.5)	85.7 (79.6–90.2)	72.6 (65.3–78.8)	90.6 (85.0–94.2)
Sensitivity	86.8 (77.4–92.7)	86.4 (77.3–92.2)	96.2 (89.4–98.7)	89.3 (80.9–94.3)	70.7 (59.6–79.8)	82.5 (72.7–89.3)	97.6 (91.5–99.3)	91.1 (82.8–95.6)
Specificity	79.3 (69.6–86.5)	85.1 (76.1–91.1)	70.5 (60.2–79.0)	83.3 (74.3–89.6)	87.9 (79.6–93.1)	88.6 (80.3–93.7)	47.6 (37.1–58.2)	90.0 (81.5–94.8)
PPV	78.6 (68.7–86.0)	84.3 (75.0–90.6)	74.5 (65.3–82.0)	83.3 (74.3–89.6)	82.8 (71.8–90.1)	86.8 (77.4–92.7)	65.0 (56.3–72.9)	90.0 (81.5–94.8)
NPV	87.3 (78.2–93.0)	87.1 (78.3–92.6)	95.4 (87.3–98.4)	89.3 (80.9–94.3)	78.4 (69.5–85.3)	84.8 (76.1–90.7)	95.1 (83.9–98.7)	91.1 (82.8–95.6)
Intra-rater agreement (Cohen κ)	0.81		0.95		0.63

**Table 4:** Cohen κ for different rater combinations for non-AI-supported readings (**a**), Cohen κ of different rater combinations for AI-supported readings (**b**)

	Rater 1	Rater 2	Rater 3	Rater 4
(a)				
Rater 1	-	0.74	0.68	0.61
Rater 2	0.74	-	0.60	0.74
Rater 3	0.68	0.60	-	0.42
Rater 4	0.61	0.74	0.42	-

(b)				
Rater 1	-	0.82	0.77	0.84
Rater 2	0.82	-	0.81	0.93
Rater 3	0.77	0.81	-	0.88
Rater 4	0.84	0.93	0.88	-

### AI-assisted assessment

AI-driven software analyses were available for 178 out of 180 scans. The diagnostic accuracy of the IPD software classifying scans as normal or abnormal was 86.0% with a sensitivity of 90.5% and a specificity of 81.9%. When diagnostic software results were available to the raters, the quality of the scans was assessed on average as very poor in 4.7% of the scans (range 1.1–10.0%), poor in 12.8% (range 7.2–16.7%), sufficient in 30.0% (range 12.8–36.1%), and good in 51.3% (range 36.7–77.8%). The quality of more scans was rated as good with software assistance. The percentage of scans rated as ‘unclear’ was 6.0% (range 2.2–10.7%). Diagnostic accuracy improved to 87.0%; this change was only statistically significant for raters 3 and 4 (range 85.7–90.6%, *p*-value range 0.002–0.51). Exploratory analyses suggested that the improved accuracy was largely driven by an increase in specificity from 71.3% (range 47.6–87.9%) without software assistance to 86.8% (range 83.3–90.0%) with software assistance. Meanwhile, the sensitivity remained similar, with a slight decrease from 87.8% without software assistance to 87.3% (range 82.5–91.1%) with software assistance. For the scans with a quality rated as sufficient or good, diagnostic accuracy was 87.6% (range 85.4–91.4%). The inter-rater agreement improved to almost perfect, with a Fleiss κ of 0.86 (95% CI 0.79–0.92), with a Cohen κ ranging from substantial to almost perfect (Cohen κ range 0.77–0.93).

## Discussion

Visual assessment of nigrosome-1 on SMWI showed high diagnostic accuracy with substantial inter-rater agreement in patients with CUPS. The addition of AI-driven software assistance improved both diagnostic accuracy and inter-rater consistency, suggesting that visual assessment of SMWI, especially when software assistance is available, may serve as a candidate MRI-based alternative to DAT imaging; these findings warrant confirmation in future prospective studies.

The diagnostic accuracy of SMWI visual assessment was comparable to that in previous studies in which a diagnostic accuracy ranging from 71.7 to 97.7% was found [12, 13, 22]. In the present study, we found a lower diagnostic accuracy than a recent multicenter study that reported an accuracy of 97.7% [11]. However, all previous studies included patients with an established diagnosis and excluded CUPS patients, as well as patients with other neurological conditions or structural lesions on the MRI. As such, these studies are not representative of the complex characteristics of a CUPS cohort that would benefit most from improved diagnostics and better reflect clinical reality. In contrast to previous studies, in the current study, we did not exclude patients based on medical history or structural brain lesions. The slightly lower diagnostic accuracy of SMWI found in the present study compared to some previous studies might therefore be attributed to the included population. Additionally, the inter-rater agreement (Fleiss κ = 0.65) reported in this study was lower than in previous research in which an inter-rater agreement with a κ value between 0.91 and 0.97 was reported [10–12, 14]. This might be the result of the included population as well. Furthermore, the agreement improved when software assistance was available. A non-diagnostic (“unclear”) rating was given in 8.3% of unaided reads and 6.0% with software assistance; such scans require follow-up or additional imaging rather than yielding a definitive result. To our knowledge, this is the first study to evaluate SMWI in a clinically uncertain parkinsonian syndrome population and assess radiologist performance together with AI-assisted interpretation in this setting.

AI-driven software assistance increased both diagnostic accuracy and inter-rater agreement. With software support, accuracy increased to 87.0%, due to an increase in specificity when software assistance was available. The benefit of software assistance was largest for the reader with the lowest unaided specificity, who tended to overclassify scans as abnormal (specificity 47.6%, 43 false positives). With assistance, this reader’s specificity rose to 90.0% with only a modest decrease in sensitivity, whereas readers with already high unaided specificity changed less. This pattern suggests that software assistance is particularly helpful for “sensitive” readers and improves consistency across readers. The inter-rater agreement reached an almost perfect level (Fleiss κ = 0.86). In addition, fewer scans were designated as “unclear.” These improvements may reflect the strong standalone performance of the software. Although the software performed well independently, in clinical practice it would most likely be implemented as assistance to radiologist visual assessment rather than as a standalone tool. The findings in this study highlight the potential added value of AI-driven software to improve both accuracy and consistency in the diagnosis of patients with CUPS. A recent study demonstrated that the impact of AI assistance varies across readers and tasks and cannot be reliably predicted by clinical experience or familiarity with AI tools [23]. These findings demonstrate the need for context-specific validation of AI applications. The consistent increase in diagnostic accuracy and inter-rater agreement observed across raters in our study suggests that SMWI interpretation may be well-suited to AI-assisted evaluation in routine clinical practice.

In this study on CUPS, we reported limited differences in the symptom assessment scales for participants with neurodegenerative and non-neurodegenerative parkinsonism. Interestingly, both groups demonstrated mean MoCA scores below the cutoff for cognitive impairment, which may have contributed to the diagnostic uncertainty observed in this population. The clinical scores tended to be worse in the non-neurodegenerative group than in the group of patients with neurodegenerative parkinsonism; however, except for the MDS-UPDRS part I, these differences did not reach statistical significance. The similarity of the symptom assessment scores, in particular for the motor symptoms, might be explained by the continued symptomatic treatment, including levodopa during symptom evaluation. The lack of significant differences in scores on the clinical questionnaires and the symptom assessment scales underscores the challenge of establishing a certain diagnosis based on clinical symptoms only in these patients.

In CUPS patients, diagnosing whether parkinsonism has a neurodegenerative cause represents an important clinical challenge. Our findings indicate that SMWI can contribute to this distinction, similar to presynaptic nuclear imaging techniques such as DAT-SPECT. Because MRI is already part of routine diagnostic workup, integrating SMWI could reduce reliance on DAT-SPECT and expand diagnostic options, particularly in centers where nuclear imaging is limited. A second diagnostic challenge in CUPS is the differentiation between PD and atypical forms of neurodegenerative parkinsonism, such as multiple system atrophy. Only a small number of patients with atypical parkinsonism were included in our study, limiting conclusions regarding this subgroup. A previous study including 8 patients with multiple system atrophy and 8 patients with progressive supranuclear palsy reported an abnormal SMWI result for 15 out of 16 scans [11]. Given the current understanding of nigrosome-1 imaging, the diagnostic value of SMWI in distinguishing atypical parkinsonism from PD is likely limited.

A limitation of our study is the retrospective selection of patients with CUPS. All patients underwent a DAT-SPECT scan before inclusion in the study. This retrospective approach allowed us to include a large cohort of CUPS patients, necessary for evaluating SMWI in a clinical setting. However, this resulted in a substantial and variable delay between DAT-SPECT and the study MRI, with a mean of 1.8 years and a range up to 4.5 years. In a progressive neurodegenerative condition such as PD, this interval may have influenced imaging findings and may affect the validity of the comparison between the index test and the reference standard. To assess this, we performed an interval-stratified sensitivity analysis (≤ 1 *versus* > 1 year), which showed no evidence that a longer interval inflated diagnostic performance. A related consequence of this design is that, by the time of the research MRI, the clinical diagnosis had often become increasingly certain over follow-up, so patients might no longer be uncertain in the strict sense at the moment of imaging. Given the absence of longitudinal SMWI studies, the impact of disease progression on diagnostic accuracy remains uncertain. Future prospective studies imaging CUPS patients at the time of diagnostic uncertainty are warranted to investigate the diagnostic value of SMWI as an important next step. A further limitation is that all MRI scans were acquired on a single 3-T scanner with a fixed acquisition and reconstruction protocol, chosen to minimize technical variability in this first evaluation of SMWI in a CUPS cohort. This nonetheless limits generalizability, as SMWI performance may depend on scanner vendor, acquisition parameters, and reconstruction pipeline. Our findings, therefore, require validation across systems and centers. Another limitation relates to reader training and experience. Because SMWI is a novel sequence, the radiologists involved in this study had no experience with the sequence prior to the study. While none of the raters reported feeling insufficiently prepared, it remains possible that a more extensive training protocol could have influenced diagnostic performance. Additionally, the unaided and software-assisted sessions were not separated by a defined washout interval. Although each case was reassessed only after the remainder of the series had been read, there could be a recall bias. In this study, the reference standard was the final diagnosis by a neurologist incorporating the DAT-SPECT results. Since postmortem pathological confirmation was not feasible in this study, the chosen reference provides a reliable alternative, given the high diagnostic accuracy of DAT-SPECT up to 98% in distinguishing neurodegenerative from non-neurodegenerative parkinsonism [1]. However, as visual SMWI assessment and DAT-SPECT scan analyses both examine possible nigrostriatal degeneration, this may have increased the apparent agreement between the visual reads and the reference standard, although the visual reads themselves did not contribute to the diagnosis.

## Conclusion

In conclusion, SMWI demonstrated high diagnostic accuracy and substantial inter-rater agreement for differentiating between neurodegenerative and non-neurodegenerative parkinsonism in a representative cohort of CUPS patients. Diagnostic performance further improved with the addition of AI software assistance, enhancing both accuracy and consistency across readers. These findings support the potential role of SMWI, particularly when combined with diagnostic software, as a diagnostic tool in the clinical evaluation of patients with CUPS. Prospective studies are warranted to validate the diagnostic value and assess its integration into routine clinical practice.

## Supplementary information

**Additional file 1:** **Supplemental figure 1.** An example of the results of the IPD software, classifying the nigrosome-1 as normal or abnormal for the left and right side. **Supplemental figure 2.** An example of the results of the NI software, providing a volumetric measurement of the nigrosome-**1** and z-score.

## Data Availability

Anonymized data and analysis scripts are available from the corresponding author upon reasonable request, subject to institutional data-sharing agreements.

## Abbreviations

AI: Artificial intelligence
BDI: Beck Depression Inventory II
CUPS: Clinically uncertain parkinsonian syndrome
DAT: Dopamine transporter
FP-CIT: Fluoropropyl-carbomethoxy-iodotropane
MDS-UPDRS: Movement Disorder Society Unified Parkinson’s Disease Rating Scale
MoCA: Montreal Cognitive Assessment
MRI: Magnetic resonance imaging
PD: Parkinson’s disease
QSM: Quantitative susceptibility mapping
SMWI: Susceptibility map-weighted imaging
SN: Substantia nigra
SPECT: Single-photon emission computed tomography
SWI: Susceptibility-weighted imaging

## References

[1] Suwijn SR, van Boheemen CJ, de Haan RJ, Tissingh G, Booij J, de Bie RM (2015) The diagnostic accuracy of dopamine transporter SPECT imaging to detect nigrostriatal cell loss in patients with Parkinson’s disease or clinically uncertain parkinsonism: a systematic review. EJNMMI Res 5:12. 10.1186/s13550-015-0087-1 PMC4385258 25853018

[2] Nam Y, Gho SM, Kim DH, Kim EY, Lee J (2017) Imaging of nigrosome 1 in substantia nigra at 3T using multiecho susceptibility map-weighted imaging (SMWI). J Magn Reson Imaging 46:528–536. 10.1002/jmri.25553 27859983

[3] Gho SM, Liu C, Li W et al (2014) Susceptibility map-weighted imaging (SMWI) for neuroimaging. Magn Reson Med 72:337–346. 10.1002/mrm.24920 24006248

[4] Blazejewska AI, Schwarz ST, Pitiot A et al (2013) Visualization of nigrosome 1 and its loss in PD: pathoanatomical correlation and *in vivo* 7 T MRI. Neurology 81:534–540. 10.1212/WNL.0b013e31829e6fd2 PMC3775686 23843466

[5] Wang JY, Zhuang QQ, Zhu LB et al (2016) Meta-analysis of brain iron levels of Parkinson’s disease patients determined by postmortem and MRI measurements. Sci Rep 6:36669. 10.1038/srep36669 PMC5101491 27827408

[6] Friedrich I, Reimann K, Jankuhn S et al (2021) Cell specific quantitative iron mapping on brain slices by immuno-microPIXE in healthy elderly and Parkinson’s disease. Acta Neuropathol Commun 9:47. 10.1186/s40478-021-01145-2 PMC7986300 33752749

[7] Sun H, Walsh AJ, Lebel RM et al (2015) Validation of quantitative susceptibility mapping with Perls’ iron staining for subcortical gray matter. Neuroimage 105:486–492. 10.1016/j.neuroimage.2014.11.010 25462797

[8] Zheng W, Nichol H, Liu S, Cheng YC, Haacke EM (2013) Measuring iron in the brain using quantitative susceptibility mapping and x-ray fluorescence imaging. Neuroimage 78:68–74. 10.1016/j.neuroimage.2013.04.022 PMC3843006 23591072

[9] Langkammer C, Schweser F, Krebs N et al (2012) Quantitative susceptibility mapping (QSM) as a means to measure brain iron? A post mortem validation study. Neuroimage 62:1593–1599. 10.1016/j.neuroimage.2012.05.049 PMC3413885 22634862

[10] Bae YJ, Song YS, Choi BS, Kim JM, Nam Y, Kim JH (2021) Comparison of susceptibility-weighted imaging and susceptibility map-weighted imaging for the diagnosis of Parkinsonism with nigral hyperintensity. Eur J Radiol 134:109398. 10.1016/j.ejrad.2020.109398 33264728

[11] Sung YH, Kim JS, Yoo SW et al (2022) A prospective multi-centre study of susceptibility map-weighted MRI for the diagnosis of neurodegenerative parkinsonism. Eur Radiol 32:3597–3608. 10.1007/s00330-021-08454-z 35064313

[12] Sung YH, Lee J, Nam Y et al (2019) Initial diagnostic workup of parkinsonism: dopamine transporter positron emission tomography *versus* susceptibility map-weighted imaging at 3T. Parkinsonism Relat Disord 62:171–178. 10.1016/j.parkreldis.2018.12.019 30580909

[13] Welton T, Hartono S, Lee W et al (2024) Classification of Parkinson’s disease by deep learning on midbrain MRI. Front Aging Neurosci 16:1425095. 10.3389/fnagi.2024.1425095 PMC11369979 39228827

[14] Shin DH, Heo H, Song S et al (2021) Automated assessment of the substantia nigra on susceptibility map-weighted imaging using deep convolutional neural networks for diagnosis of Idiopathic Parkinson’s disease. Parkinsonism Relat Disord 85:84–90. 10.1016/j.parkreldis.2021.03.004 33761389

[15] Suh PS, Heo H, Suh CH et al (2025) Deep learning-based algorithm for automatic quantification of nigrosome-1 and parkinsonism classification using susceptibility map-weighted MRI. AJNR Am J Neuroradiol 46:999–1006. 10.3174/ajnr.A8585 PMC12091983 39547802

[16] Wallert ED, van de Giessen E, Beudel M et al (2026) Automated assessment of susceptibility map-weighted imaging in patients with clinically uncertain parkinsonism. Parkinsonism Relat Disord 144:108198. 10.1016/j.parkreldis.2026.108198 41579429

[17] Morbelli S, Esposito G, Arbizu J et al (2020) EANM practice guideline/SNMMI procedure standard for dopaminergic imaging in Parkinsonian syndromes 1.0. Eur J Nucl Med Mol Imaging 47:1885–1912. 10.1007/s00259-020-04817-8 PMC7300075 32388612

[18] Postuma RB, Berg D, Stern M et al (2015) MDS clinical diagnostic criteria for Parkinson’s disease. Mov Disord 30:1591–1601. 10.1002/mds.26424 26474316

[19] Hoehn MM, Yahr MD (1967) Parkinsonism: onset, progression and mortality. Neurology 17:427–442. 10.1212/wnl.17.5.427 6067254

[20] Beck AT, Steer RA, Brown GK (1996) Manual for the Beck Depression Inventory-II. Psychological Corporation, San Antonio

[21] Nasreddine ZS, Phillips NA, Bedirian V et al (2005) The Montreal Cognitive Assessment, MoCA: a brief screening tool for mild cognitive impairment. J Am Geriatr Soc 53:695–699. 10.1111/j.1532-5415.2005.53221.x 15817019

[22] Bae YJ, Song YS, Kim JM et al (2021) Determining the degree of dopaminergic denervation based on the loss of nigral hyperintensity on SMWI in parkinsonism. AJNR Am J Neuroradiol 42:681–687. 10.3174/ajnr.A6960 PMC8041011 33509919

[23] Yu F, Moehring A, Banerjee O, Salz T, Agarwal N, Rajpurkar P (2024) Heterogeneity and predictors of the effects of AI assistance on radiologists. Nat Med 30:837–849. 10.1038/s41591-024-02850-w PMC10957478 38504016

