# Supplementary material for: Diagnostic accuracy and reliability of susceptibility map-weighted imaging in patients with clinically uncertain parkinsonian syndromes
Source: Eur Radiol Exp. 2026 Nov 14;10:184. doi: 10.1186/s41747-026-00832-w (PMC13769795; doi:10.1186/s41747-026-00832-w)

Diagnostic Accuracy and Reliability of Susceptibility Map-Weighted Imaging in Patients with Clinically Uncertain Parkinsonian Syndromes

Electronic Supplementary Material

Supplemental figure 1. An example of the results of the IPD software, classifying the nigrosome-1 as normal or abnormal for the left and right side.

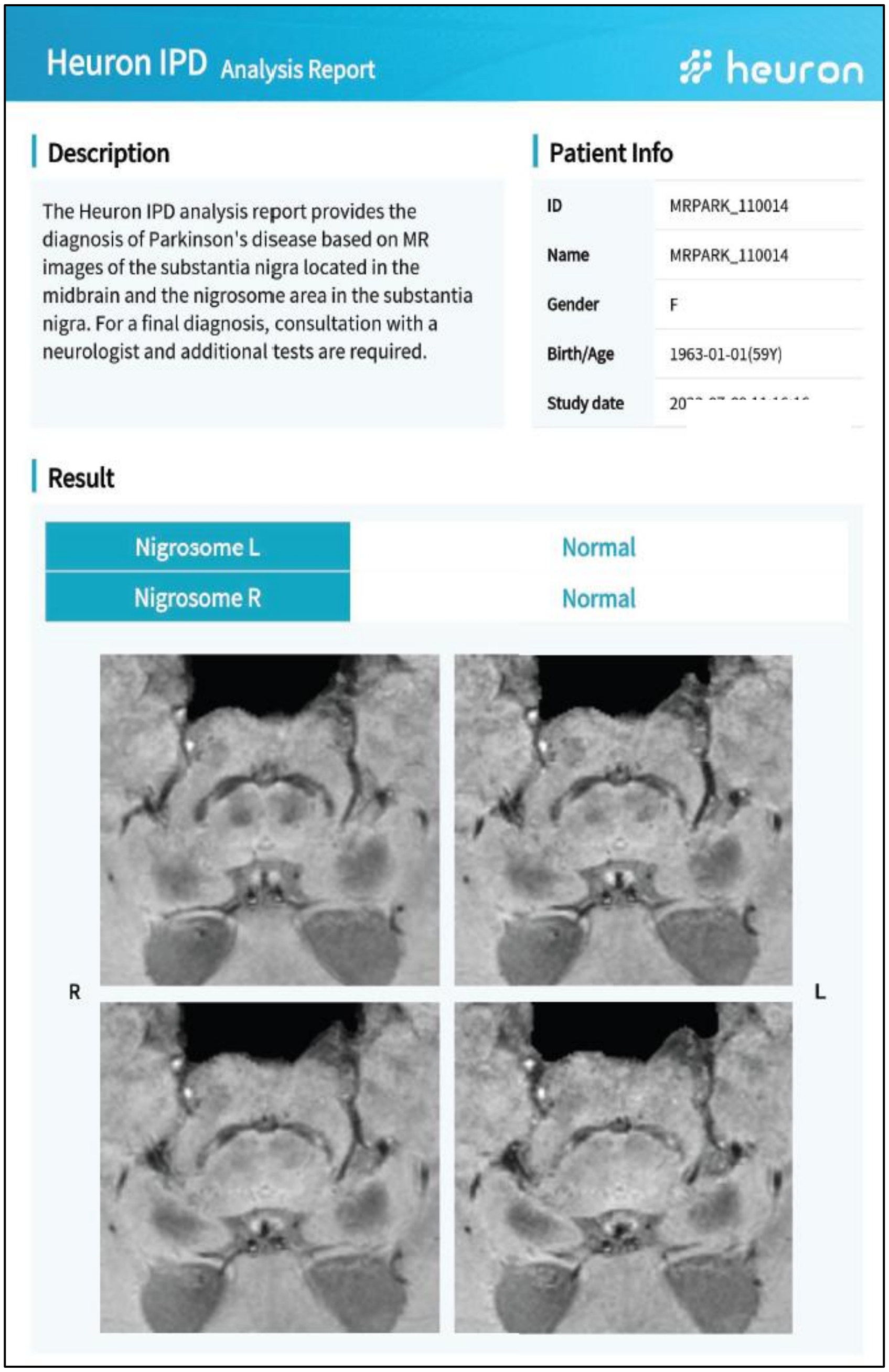

Supplemental figure 2 An example of the results of the NI software, providing a volumetric measurement of the nigrosome-1 and z-score.

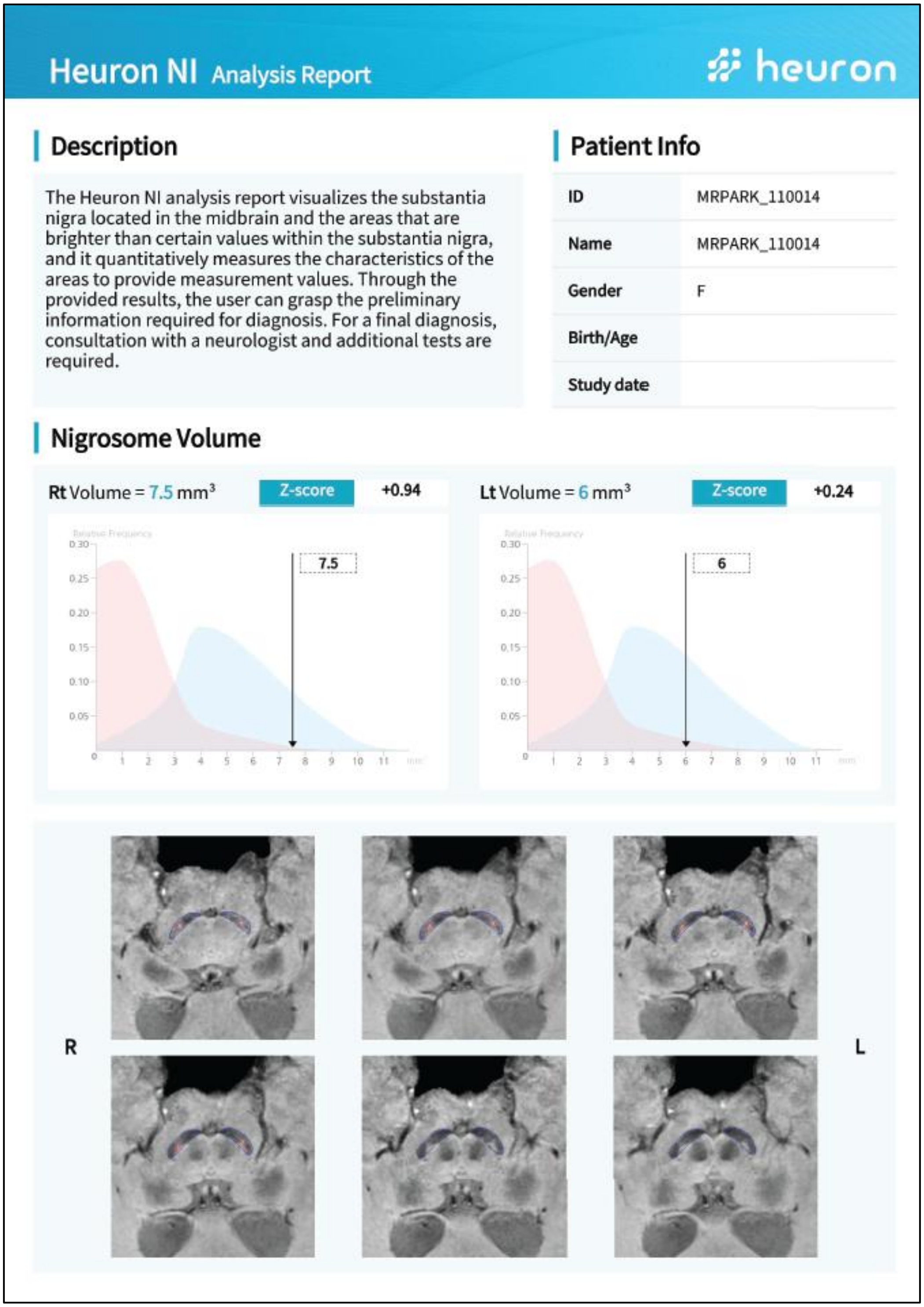

Supplement: Supplementary file 1 [file 41747_2026_832_MOESM1_ESM.pdf]
